# Power Calculation of Multi-step Combined Principal Components with Applications to Genetic Association Studies

**DOI:** 10.1038/srep26243

**Published:** 2016-05-18

**Authors:** Zhengbang Li, Wei Zhang, Dongdong Pan, Qizhai Li

**Affiliations:** 1 School of Mathematics and Statistics & Hubei Key Laboratory of Mathematical Sciences, Central China Normal University, Wuhan, 430079, China; 2 Academy of Mathematics and Systems Science, Chinese Academy of Sciences, Beijing, 100190, China; 3 Department of Statistics, Yunnan University, Kunming, 650091, China

## Abstract

Principal component analysis (PCA) is a useful tool to identify important linear combination of correlated variables in multivariate analysis and has been applied to detect association between genetic variants and human complex diseases of interest. How to choose adequate number of principal components (PCs) to represent the original system in an optimal way is a key issue for PCA. Note that the traditional PCA, only using a few top PCs while discarding the other PCs, might significantly lose power in genetic association studies if all the PCs contain non-ignorable signals. In order to make full use of information from all PCs, Aschard and his colleagues have proposed a multi-step combined PCs method (named mCPC) recently, which performs well especially when several traits are highly correlated. However, the power superiority of mCPC has just been illustrated by simulation, while the theoretical power performance of mCPC has not been studied yet. In this work, we attempt to investigate theoretical properties of mCPC and further propose a novel and efficient strategy to combine PCs. Extensive simulation results confirm that the proposed method is more robust than existing procedures. A real data application to detect the association between gene TRAF1-C5 and rheumatoid arthritis further shows good performance of the proposed procedure.

Identification of genetic variants associated with human complex diseases can help investigators further understand genetic structure of diseases of interest. Compared with single-marker analysis, which tests every marker individually and is commonly employed in genome-wide association study, multiple-marker test has been well appreciated because of its potentially improved statistical power. Statistical methods for multiple-marker analysis can be summarized as synthesizing single-marker test statistics such as Hotelling’s *T*^2^ test^1^,^2^,^3^ and summation of squared univariate test^4^,^5^, weighted Fourier transformation^6^, variance-components score test^7^, principal components regression method^8^,^9^,^10^, and Kernel-machine-based test^11^. Performances of these methods have been explored by intensive computer simulations^1^,^12^,^13^. Their results showed that when the number of SNPs is relatively large, variance-component-based methods and principal components regression methods were found to have competitive power.

As it is well known that principal component analysis (PCA) is a useful tool to search for important characteristics among correlated variables. A key issue in developing an effective PCA model is choosing an adequate number of principal components (PCs) to represent the system in an optimal way. Taking advantage of the size of variances, Hocking^14^ provided a firm rule for retaining PCs in the framework of regression models. Usually, in PCA, investigators only used a few top principal components and discarded the other PCs. Recently, some investigators have illustrated that commonly used method for choosing PCs is not always reasonable. In fact, as early as in 1982, Jollife^15^ showed an interesting counter-intuitive phenomenon that principal components explaining a small amount of variances can be as important as those explaining a large amount of variances when analyzing non-genetic data. Aschard *et al.*^16^ confirmed this phenomenon when analyzing genetic data and proposed a called multi-step combined principal component (mCPC) strategy. However, the performance of mCPC strongly depends on how to partition all PCs.

Without loss of generality, suppose a random vector *T* follows a multivariate normal distribution with a *m* × 1 mean vector *μ* and known *m* × *m* covariance matrix *V*. We want to test the null hypothesis *H*_0_: *μ* = 0. Therefore, a Chi-squared statistic can be used for testing *H*_0_. However, when *m* is large, which is fairly common in genome-wide association studies, Chi-squared test might substantially lose power due to its large degrees of freedom. To reduce degrees of freedom, PCA is recommended. Based on orthogonal decomposition, we have *V* = *Q*Λ*Q*^*τ*^, where 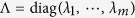 with 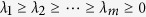, 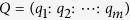, and *q*_*i*_ is called as the eigenvector corresponding to the eigenvalue *λ*_*i*_, 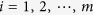. Define 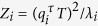 for 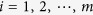. We note that *Z*_*i*_ is related to the *i*th PC for 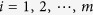. Under *H*_0_, 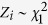, a central Chi-squared distribution with 1 degree of freedom for 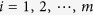. Under the alternative hypothesis, 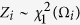, a noncentral Chi-squared distribution with 1 degree of freedom and non-centrality parameter 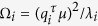, for 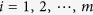.

For simplicity with *m* = 2, we consider a linear model with a normally distributed phenotype *Y*, which depends on two scaled genotypes *G*_1_ and *G*_2_ that are also normally distributed with mean 0 and variance 1. So the phenotype can be expressed as: *Y* = *β*_0_ + *G*_1_*β*_1_ + *G*_2_*β*_2_ + *ε*, where *ε* is the random error term which is distributed from the standard normal distribution. For this general model, the principal components of these two genotypes are 
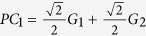
, and 
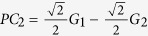
. By some algebras, we can get 
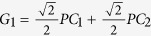
, and 
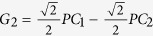
. Phenotype *Y* can be reexpressed as: 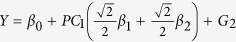

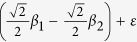
, which indicates that *PC*_2_ may be very important as 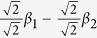 is large, although the variance of *PC*_2_ is less than that of *PC*_1_. So we can not discard any PCs arbitrarily. In order to test *H*_0_: *μ* = 0, Aschard *et al.*^17^ proposed a multi-step combined principal component (mCPC) as following 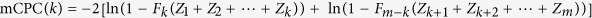, where *F*_*k*_ (·) is cumulative distribution function of a central Chi-squared random variable with *k* degrees of freedom. Moreover, they used simulation to compare the power of various PCA-based strategies when analyzing up to 100 correlated traits, and showed that their method with combining the signals across all PCs could have greater power. However, there has not been an in-depth study of the theoretical properties of mCPC in Aschard *et al.*’s paper^16^. Obviously, Aschard *et al.*^16^ find an unusual way to fully utilize all PCs. Another key issue is to decide the value of *k*. A commonly used method for selecting *k* is based on cumulative contribution rates, which are equal to 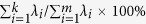, and denoted by *c*_*k*_ for 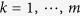, respectively. Let 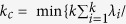

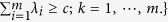
, for any *c* ∈ [0, 1]. Aschard *et al.*^16^ followed the traditional way to use mCPC (*k*) with *k* being determined by cumulative contribution rate of 80%.

In this work, we focus on the theoretical power of mCPC and find that the maximum power of mCPC is related to the maximum noncentral parameters under alternative hypothesis. We also find that the noncentral parameter corresponding to the top PC (the first PC which corresponds to the largest eigenvalue) is greater than 0 under most scenarios and those of other PCs do not possess this property when only a few means of all PCs are non-zero under alternative and the correlation coefficients among original variables are relatively large. Herein we propose a method tCPC. Based on numerical results, the tCPC is more powerful than the existing procedures under most of the considered scenarios.

## Results

### Theoretical Properties of mCPC (*k*)

For the multiple genetic variants association studies, the above random vector can be written as 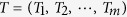, where *T*_*i*_ is the statistic that is used to test for the association between the phenotype of interested and the *i*th genetic variants, 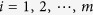. *V* is the covariance matrix of the random vector *T*. Through the eigen-decomposition of the covariance matrix, we have *V* = *Q*Λ*Q*^*τ*^, where 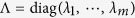 with 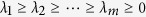, 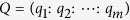, and *q*_*i*_ is the eigenvector corresponding to the eigenvalue *λ*_*i*_, 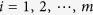. Then we can obtain transformed statistics as 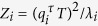, 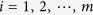. Furthermore, under *H*_0_, *Z*_*i*_ follows a central Chi-squared distribution with 1 degree of freedom for 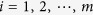. Under the alternative hypothesis, 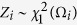, a noncentral Chi-squared distribution with 1 degree of freedom and non-centrality parameter Ω_*i*_, 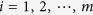.

For 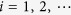, let 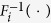 be the inverse function of *F*_*i*_ (·). Note that for any given *x* ∈ [0, 1], 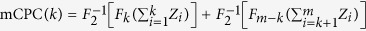. Under *H*_0_, both 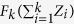, and 
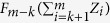
 follow uniform distribution on [0, 1] and they are independent to each other. So both 
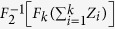
, and 
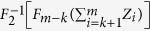
 follow Chi-square distributions with 2 degrees of freedom, then mCPC (*k*) follows a central Chi-squared distribution with 4 degrees of freedom.

According to Sankaran^17^, probability density function of a noncentral Chi-squared distribution with *d* degrees of freedom and non-centrality parameter *ξ* is 
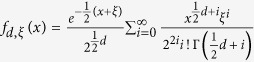
. Denote 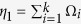, and 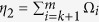. For any *x* > 0, and 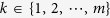, the probability density function of 
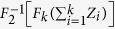
 is


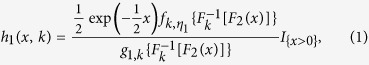


where 
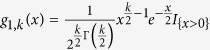
, and the probability density function of 
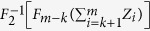
 is


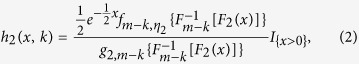


with 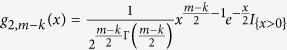.

Let *C*_1−*α*_ be 1 − *α* quantile of a central Chi-squared distribution with 4 degrees of freedom. The power of mCPC (*k*) under the significance level *α* is


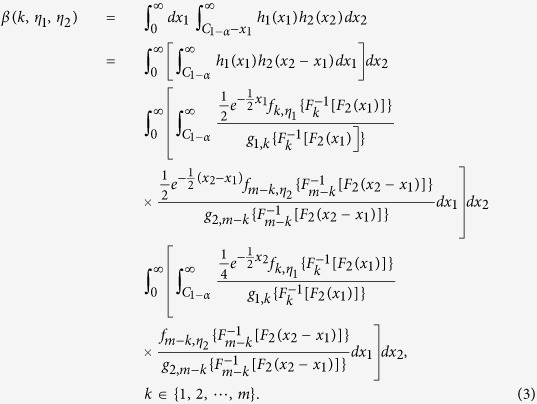


Based on the above notations, the non-centrality parameter of the distribution of *Z*_1_ which corresponds to the first PC is 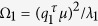, where *μ* is the mean vector of *T* under the alternative hypothesis. Ω_1_ = 0 if and only if the mean vector *μ* belongs to the space that expanded by the other *m* − 1 eigenvectors 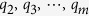, that is 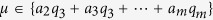, 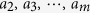 are *m* − 1 real numbers. However, for a *m*-dimensional space, 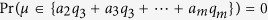. Hence, the non-centrality parameter of the Chi-squared distribution of the statistic *Z*_1_ is not equal to 0 almost everywhere. Besides this, and since the top PC possesses the largest variation among all PCs and *k* = 1 is a boundary point of the set consisting of 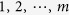, herein we propose to use the following strategy (named tCPC) to combine all PCs


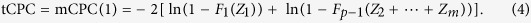


Under null hypothesis of no association at any locus, tCPC follows a central Chi-squared distribution with 4 degrees of freedom.

### Simulation Settings and Numerical Results

In this subsection, we conduct simulation studies to compare powers between tCPC to some exiting approaches such as Hotelling’s *T*^2^ test (HT)^1^,^2^,^3^, ordinary PCA 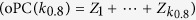, summation of squared univariate test statistic (SSU)^4^, sequence kernel association test (SKAT)^11^ and multi-step combine principal component test mCPC (*k*_0.8_)^17^.

Consider testing association between *m* genetic variants (or SNPs) and a complex human disease. Let 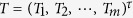 be a test statistic, where *τ* means the transpose of a vector or matrix. For example, we can construct *T* using the method in Chatterjee *et al.*^18^ to detect genetic association between *m* SNPs and a binary trait as


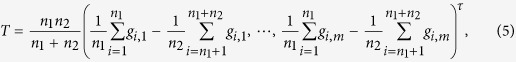


where *g*_*i*,*j*_ denotes the genotype of the *j*th SNP for the *i*th individual and *n*_1_ and *n*_2_ are the sample size of the case group and control group, respectively. Under the null hypothesis that these *m* SNPs are not associated with the disease of interest, *T* follows a multivariate normal distribution *N* (*μ*_*m*×1_, *V*_*m*×*m*_) asymptotically with the mean vector *μ*_*m*×1_ and covariance matrix 
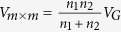
, in which *V*_*G*_ is the pooled-sample covariance matrix of all SNPs.

In order to obtain *T* and *V*_*m*×*m*_, we first generate a latent vector with length of 20 from a multivariate normal distribution with covariance structures of a compound symmetry with equal pairwise correlation *ρ*. Then, this latent vector is dichotomized to yield a haplotype with predesignated minor allele frequency (MAF). We repeat the above process 100,000 times to form a large population. Without loss of generality, we designate the first SNP as disease-causal SNP with MAF being *p*, and other SNPs as noncausal SNPs with MAFs all being *q*. Both sizes of case samples and control samples are set to be 1,000. Case or control status of one subject is generated from a logistic regression model


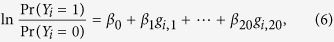


with *β*_0_ = −1.5, *β*_1_ ∈ {ln (1.4), ln (1.6)} denoting the log odds ratio for the disease-causal SNP, and 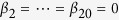 denoting log odds ratios for the non-causal SNPs, where *Y*_*i*_ = 1 or 0 represents disease or healthy status of the *i*th individual, 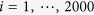. The nominal significance level is 0.05 throughout the whole simulation, and the number of replicates is 1,000. All parameter settings and their relevant results are displayed in Table 1. Table 1 shows that all these tests can control type I error rate correctly. For example, when the correlation coefficient of these 20 SNPs are equal to *ρ* = 0.50, *β*_1_ = 0 and *p* = *q* = 0.20, the empirical type I error rates of HT, oPC (*k*_0.8_), SSU, SKAT, mCPC (*k*_0.8_), and tCPC are 0.051, 0.052, 0.043, 0.049, 0.051, and 0.045, respectively. The results of power comparison shows that tCPC performs more robustly than the other methods. For example, when the correlation coefficients of these 20 SNPs are uniformly equal to 0.20 and *β*_1_ = ln 1.4, *p* = *q* = 0.20, the empirical powers of HT, oPC (*k*_0.8_), SSU, SKAT, mCPC (*k*_0.8_), and tCPC are 0.754, 0.736, 0.772, 0.766, 0.700, and 0.766, respectively. The empirical power of tCPC is a little lower than that of SSU in this scenario. However, when *ρ* = 0.50, *β*_1_ = ln 1.4, and *p* = *q* = 0.20, the empirical powers of HT, oPC (*k*_0.8_), SSU, SKAT, mCPC (*k*_0.8_), and tCPC are 0.724, 0.756, 0.810, 0.816, 0.702, and 0.856, respectively. It is obvious that tCPC performs the best among all the considered procedures in this setting.

Next we consider a decreasing correlation structure. As a preliminary step, a latent vector with length of 20 is generated from a multivariate normal distribution with covariance matrix being (*ρ*^|*i*−*j*|^)_20×20_. Other simulation settings are similar as above and are shown in Table 2. As presented in Table 2, the empirical powers of all the tests are close to the nominal significance level which indicates that they can control type I error rate correctly. For instance, when *ρ* = 0.50, *β*_1_ = 0 and *p* = *q* = 0.20, the empirical type I error rates of HT, oPC (*k*_0.8_), SSU, SKAT, mCPC (*k*_0.8_), and tCPC are 0.036, 0.032, 0.04, 0.044, 0.038, and 0.042, respectively. For power comparison, tCPC still performs more robustly than the other methods. For example, when correlations of these 20 SNPs are decreasing with distance as *ρ* = 0.80, *β*_1_ = ln 1.4, *p* = 0.20, and *q* = 0.20, the empirical powers of HT, oPC (*k*_0.8_), SSU, SKAT, mCPC (*k*_0.8_), and tCPC are 0.77, 0.846, 0.772, 0.762, 0.788, and 0.836, respectively. The empirical power of tCPC is a little lower than that of oPC (*k*_0.8_) in this case. However, when *ρ* = 0.95, *β*_1_ = ln 1.4, *p* = 0.20, and *q* = 0.20, the powers of HT, oPC (*k*_0.8_), SSU, SKAT, mCPC (*k*_0.8_) and tCPC are 0.746, 0.898, 0.858, 0.858, 0.86 and 0.864, respectively. It indicates that tCPC gives the maximum power among the six methods in this scenario. Compared Tables 1 and 2 comprehensively, we can see that, when linkage disequilibrium extents among all SNPs are relatively strong, tCPC performs more robustly than existing statistical methods.

### Applications to gene TRAF1-C5 associated with Rheumatoid Arthritis

We apply tCPC and the other five existing tests to detect the association between gene TRAF1-C5 and rheumatoid arthritis using the data from the Genetic Analysis Workshop 16^19^. Our goal is to detect whether there is an association between gene TRAF1-C5 and rheumatoid arthritis. This gene has been reported to be deleterious previously^20^. There are 2,062 subjects including 868 cases and 1,194 controls in this study. The gene TRAF1-C5 consists of 38 SNPs. The p-values of HT, oPC (0.8), SSU, SKAT, mCPC (*k*_0.8_) and tCPC of detecting associations between gene TRAF1-C5 and rheumatoid arthritis are 5.21 × 10^−5^, 7.58 × 10^−3^, 5.95 × 10^−4^, 6.50 × 10^−5^, 7.56 × 10^−5^ and 3.75 × 10^−5^, respectively. If we use the p-value threshold of 5 × 10^−5^ as the moderate association at the genome-wide level as Burton *et al.*^21^, only the proposed tCPC can detect the moderate-strong association signal between the gene TRAF1-C5 and rheumatoid arthritis.

## Discussion

Principal component analysis is a common tool to grasp important features of correlated variables and has been applied in genetic association studies. In principal component analysis, cumulative contribution rate of 80% or 90% is commonly adopted to choose PCs. However, this adoption is not always suitable since PCs with low contribution rate might be much more strongly correlated with the outcome than those with large contribution rate. To overcome this drawback, a mCPC method was developed recently^16^. In this study, we explored theoretical powers of mCPC deeply and find out that the maximum power of mCPC depends on the maximum noncentral parameters of Chi-squared distributions for all PCs under the alternative hypothesis. However, it is difficult to obtain this information beforehand in practice. In view of this, we propose a novel and robust strategy to combine PCs. We also propose a test for genome-wide association studies and compare powers of this test to mCPC (*k*_0.8_) and some other existing procedures such as Hotelling’s *T*^2^ test (HT), oPC (*k*_0.8_) SSU and SKAT by extensive simulations. All simulation results show that our proposed procedure is more robust than mCPC, HT, oPC (*k*_0.8_), SSU and SKAT. Results of real data analysis further demonstrates good performances of our proposed test. We suggest researchers to employ our robust strategy when they consider using principal component analysis method in the future.

It should be noted that our proposed procedure is built upon test by Chatterjee *et al.*^18^ which was designed to detect association between a marker and a binary trait. It can be easily extended to other application fields. For instance, it has been used in pleiotropic genetic study to identify deleterious genetic variants associated with multiple traits^16^. In addition, our proposed test can also be used to detect the association between genetic variants and quantitative traits in framework of linear model, and ordinal traits on basis of proportional odds model. If quantitative traits do not follow normal distribution, one can consider constructing a multivariate nonparametric trend test^22^ and then employ our proposed strategy to combine them.

## Methods

### Maximum Powers of mCPC and ordinary PCA over extensive scenarios

**Figure 1:**
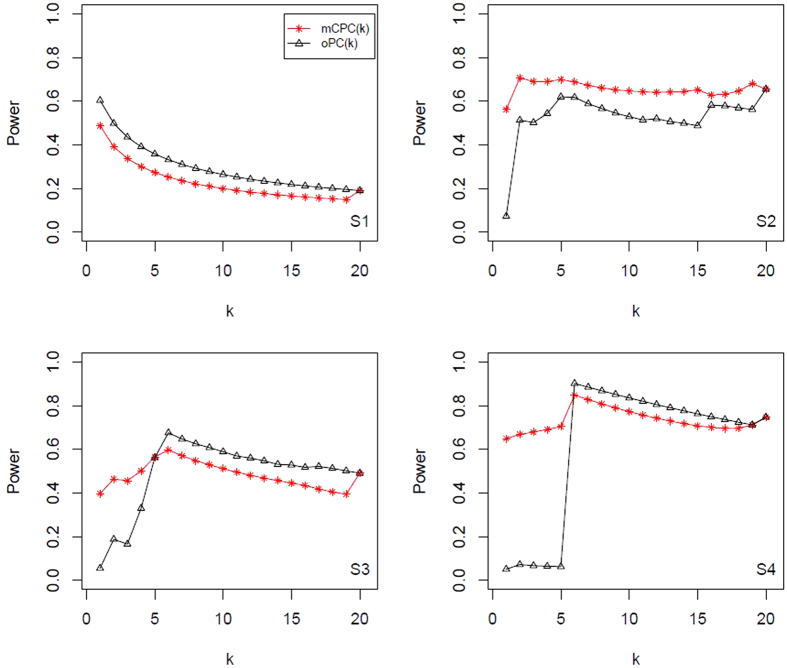
Powers of mCPC (*k*) and oPC (*k*) under significant level *α* = 0.05 for Scenarios (S1) to (S4).

**Figure 2:**
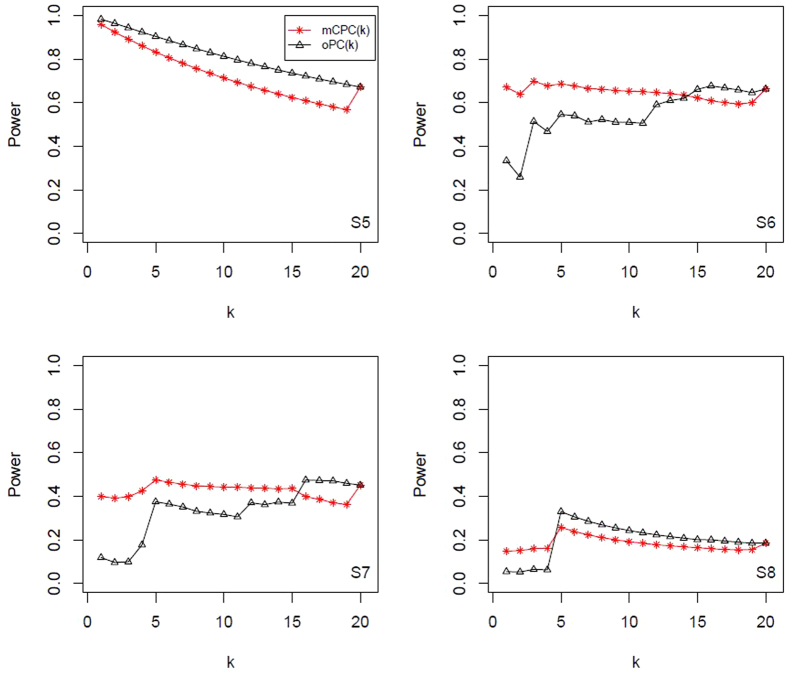
Powers of mCPC (*k*) and oPC (*k*) under significant level *α* = 0.05 for Scenarios (S5) to (S8).

**Figure 3:**
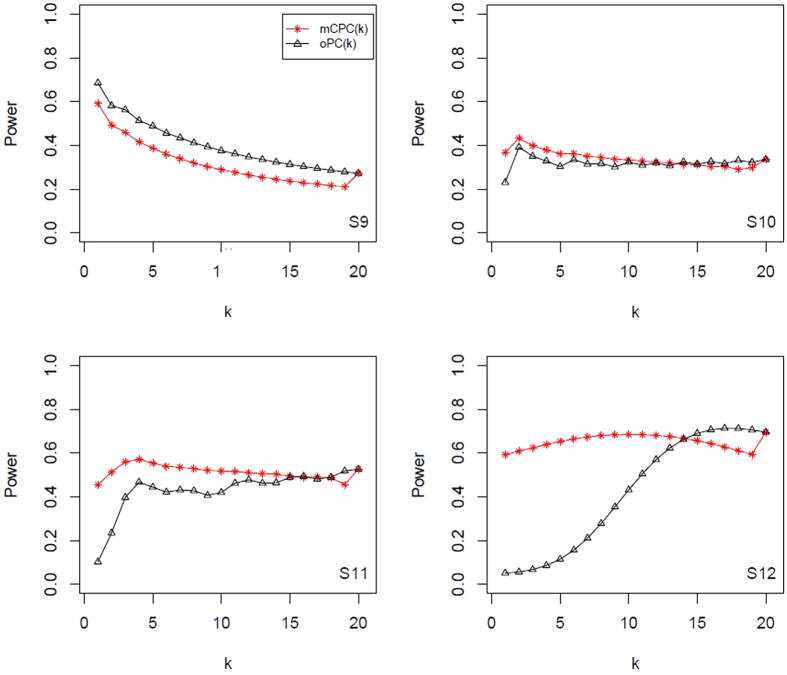
Powers of mCPC (*k*) and oPC (*k*) under significant level *α* = 0.05 for Scenarios (S9) to (S12).

**Figure 4:**
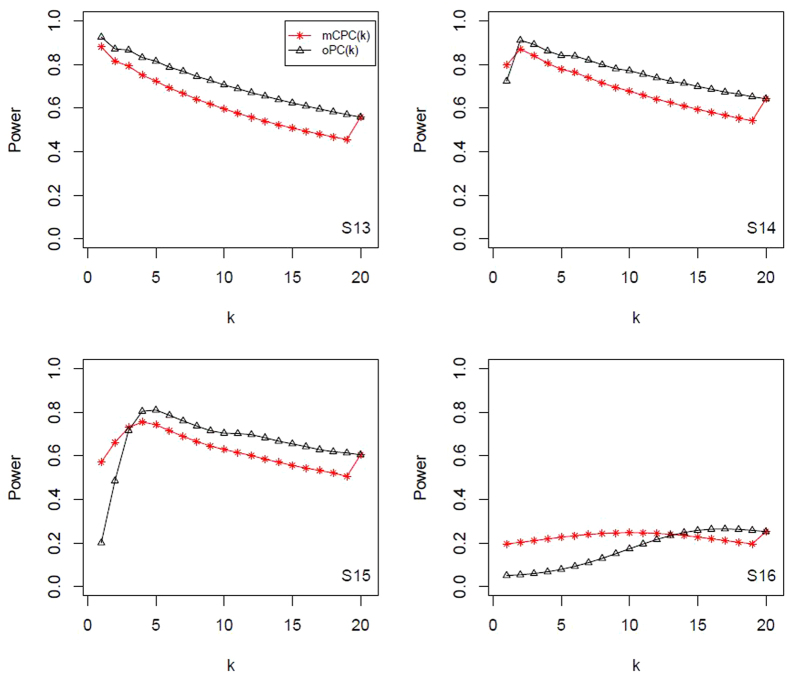
Powers of mCPC (*k*) and oPC (*k*) under significant level *α* = 0.05 for Scenarios (S3) to (S16).

For fixed *m*, the powers of mCPC (*k*) mainly depend on *k*, *μ* and *V*_*m*×*m*_. We set different mean vectors under the alternative hypothesis among different covariance matrices *V*_*m*×*m*_. We also consider two types of *V*_*m*×*m*_: one is that *m*-dimension variables are uniformly correlated, which means covariance matrix *V*_*m*×*m*_ is a symmetry positively definite matrix with diagonal elements all being 1 and non-diagonal elements all being *ρ*; the other is that all correlations among these *m* variables are decreasing considering the “physical” distance (SNP location), which means *V*_*m*×*m*_ = (*ρ*^|*i*−*j*|^)_*m*×*m*_. Without loss of generality, *ρ* is chosen to be 0.8 for strong linkage disequilibrium and 0.2 for weak linkage disequilibrium. Here, we consider *m* = 20. Note that, a test based on ordinary PCA can be gained, which is denoted by oPC (*k*) with 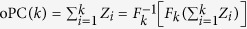, where 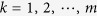. Obviously, powers of oPC (*k*) are also affected by *k* when 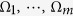 are given. In order to view powers of mCPC (*k*) and oPC (*k*) comprehensively, we set *α* to be 0.05, and calculate powers of mCPC (*k*) and oPC (*k*) by numerical integration in R software under scenarios S1 to S16. All parameter settings about scenario S1 to S16 are displayed in Table 3. We calculate eigenvalues of *V*_*m*×*m*_, *c*_*i*_, Ω_*i*_ of all scenarios S1 to S16 for 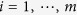, and display all results in Tables 4 and 5. All power results of mCPC and ordinary PCA are displayed in Figs 1, 2, 3, 4. Under the same correlation structure and mean vector, the powers of mCPC (*k*) and oPC (*k*) are affected strongly by selection of *k*. From Tables 3, 4, 5 and Figs 1, 2, 3, 4, we can find that both the maximum powers of mCPC (*k*) and oPC (*k*) are related to the maximum non-centrality parameters of all PCs as *k* is from 1 to 20. For example, in Scenario S15, the non-centrality parameter of the second PC is the maximum among those of all PCs, and mCPC (2) has the maximum power. In another example, under the scenario S2, the non-centrality parameter of the third PC is the maximum, mCPC (4) has the maximum power, and powers of mCPC (3) and mCPC (4) are close. We can also find out that ordinary way to select *k* to construct mCPC (*k*) is not always desirable. For example, in Scenario S12, *k*_0.8_ = 5, but oPC (5) has power as low as 0.115. It is verified that mCPC is more desirable than ordinary PCA based on Figs 1, 2, 3, 4. It is also verified that the selection of *k* according to the cumulative contribution rate is not robust. One can just follow the common adoption and choose *c* = 80% or 90% for oPC (*k*_*c*_), but it will result in loss of power substantially under some situations. Furthermore, we draw a conclusion that mCPC (*k*_*c*_) performs more robust than oPC (*k*_*c*_) similar, since mCPC (*k*_*c*_) has reasonable powers over all the considered scenarios. For example, in Scenario S2, these 20 variables are uniformly correlated with *ρ* = 0.8 and 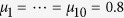, 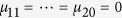, the power of oPC (*k*_0.8_) is 0.073, which is far less than the power of mCPC (*k*_*c*_), which is 0.57.

### A novel robust strategy to combine PCs

A further investigation of the maximum powers of mCPC (*k*) and oPC (*k*) shows that both of them are related to non-centrality parameters of the Chi-square distributions under the alternative hypothesis. For example, about scenario S16 in Table 5, the non-centrality parameters of all 20 PCs are 0.01, 0.05, 0.11, 0.19, 0.27, 0.37, 0.45, 0.53, 0.58, 0.61, 0.62, 0.60, 0.55, 0.48, 0.38, 0.30, 0.21, 0.12, 0.06 and 0.01 respectively, and non-centrality parameter being 0.62 which belongs to the 11th PC is the largest one among the non-centrality parameters of all 20 PCs. mCPC (10) takes the maximum power with 0.248 and the power of mCPC (11) is 0.247, which is very close to that of mCPC (10). The difference maybe are caused by numerical computing errors. The cumulative contribution rate of the top 10 PCs are 62.25%, which is much less than 80%. It is worth noted that the non-centrality parameters are determined by means and covariance matrix, which are hard to know in practice. Therefore, if we can know some prior information on means and covariance matrix, then the optimal strategy for selection of *k* become more prone to obtain. Aschard *et al.*^17^ proposed to use mCPC (*k*) with *k* being determined by cumulative contribution rate of 80%.

As shown above, using 80% cumulative contribution rate might not be a robust strategy, and it will give a very low power in some cases (e.g., Fig. 1). According to numerical results in Scenarios S1 to S16, we propose to use the tCPC method to combine all PCs.

## Additional Information

**How to cite this article**: Li, Z. *et al.* Power Calculation of Multi-step Combined Principal Components with Applications to Genetic Association Studies. *Sci. Rep.* **6**, 26243; doi: 10.1038/srep26243 (2016).

## Figures and Tables

**Table 1:** Empirical type I error rates and powers of HT, oPC (*k*_0.8_), SSU, SKAT, mCPC (*k*_0.8_) and tCPC for Constant correlations.

40.5 cmType	*ρ*	*β*_1_	*p*	*q*	HT	oPC (0.8)	SSU	SKAT	mCPC (*k*_0.8_)	tCPC
40.1 cmI 40.5 cm error	0.20	0	0.20	0.20	0.047	0.047	0.044	0.048	0.044	0.048
	0.50	0	0.20	0.20	0.051	0.052	0.043	0.049	0.051	0.045
	0.80	0	0.20	0.20	0.053	0.049	0.051	0.052	0.050	0.051
	0.95	0	0.20	0.20	0.046	0.053	0.049	0.052	0.047	0.047
	0.20	ln 1.6	0.05	0.30	0.448	0.054	0.140	0.446	0.610	0.404
	0.20	ln 1.4	0.20	0.20	0.754	0.736	0.772	0.762	0.700	0.766
	0.20	ln 1.4	0.30	0.05	0.884	0.922	0.996	0.886	0.856	0.996
	0.50	ln 1.6	0.05	0.30	0.450	0.106	0.288	0.430	0.524	0.496
40.8 cm Power	0.50	ln 1.4	0.20	0.20	0.724	0.756	0.810	0.816	0.702	0.856
	0.50	ln 1.4	0.30	0.05	0.886	0.928	0.996	0.894	0.874	0.988
	0.80	ln 1.6	0.05	0.30	0.450	0.144	0.352	0.440	0.456	0.528
	0.80	ln 1.4	0.20	0.20	0.740	0.778	0.918	0.918	0.754	0.924
	0.80	ln 1.4	0.30	0.05	0.872	0.942	0.984	0.818	0.910	0.964
	0.95	ln 1.6	0.05	0.30	0.484	0.246	0.300	0.368	0.500	0.520
	0.95	ln 1.4	0.20	0.20	0.736	0.940	0.972	0.972	0.924	0.948
	0.95	ln 1.4	0.30	0.05	0.880	0.996	0.962	0.684	0.988	0.920

**Table 2:** Empirical type-1 error rates and powers of HT, oPC (*k*_0.8_), SSU, SKAT, mCPC (*k*_0.8_) and tCPC for decreasing correlations.

40.5 cmType	*ρ*	*β*_1_	*p*	*q*	HT	oPC (0.8)	SSU	SKAT	mCPC (*k*_0.8_)	tCPC
40.1 cm I 40.5 cm error	0.20	0	0.20	0.20	0.062	0.048	0.054	0.052	0.052	0.040
	0.50	0	0.20	0.20	0.036	0.032	0.040	0.044	0.038	0.042
	0.80	0	0.20	0.20	0.052	0.038	0.042	0.044	0.056	0.046
	0.95	0	0.20	0.20	0.036	0.046	0.052	0.054	0.042	0.048
	0.20	ln 1.6	0.05	0.30	0.470	0.060	0.110	0.470	0.650	0.386
	0.20	ln 1.4	0.20	0.20	0.746	0.756	0.766	0.738	0.700	0.688
	0.20	ln 1.4	0.30	0.05	0.886	0.926	0.998	0.890	0.864	0.998
	0.50	ln 1.6	0.05	0.30	0.490	0.074	0.126	0.466	0.568	0.390
40.8 cm Power	0.50	ln 1.4	0.20	0.20	0.722	0.784	0.742	0.706	0.686	0.662
	0.50	ln 1.4	0.30	0.05	0.882	0.914	0.996	0.862	0.856	0.996
	0.80	ln 1.6	0.05	0.30	0.482	0.120	0.148	0.350	0.478	0.400
	0.80	ln 1.4	0.20	0.20	0.770	0.846	0.772	0.762	0.788	0.836
	0.80	ln 1.4	0.30	0.05	0.888	0.946	0.994	0.852	0.912	0.988
	0.95	ln 1.6	0.05	0.30	0.480	0.192	0.266	0.358	0.464	0.510
	0.95	ln 1.4	0.20	0.20	0.746	0.898	0.858	0.858	0.860	0.864
	0.95	ln 1.4	0.30	0.05	0.866	0.972	0.982	0.732	0.962	0.936

**Table 3:** Parameter settings about means and covariance matrices.

Covariance matrix	Scenarios	Mean vectors
Uniform correlation with *ρ* = 0.8	(S1)	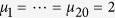
	(S2)	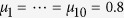 , 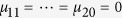
	(S3)	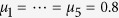 , 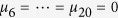
	(S4)	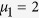 , 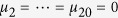
Uniform correlation with *ρ* = 0.2	(S5)	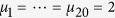
	(S6)	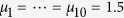 , 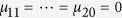
	(S7)	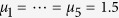 , 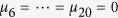
	(S8)	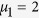 , 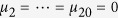
Decreasing correlation with *ρ* = 0.8	(S9)	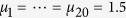
	(S10)	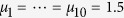 , 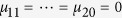
	(S11)	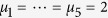 , 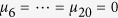
	(S12)	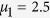 , 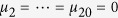
Decreasing correlation with *ρ* = 0.2	(S13)	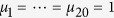
	(S14)	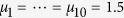 , 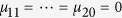
	(S15)	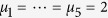 , 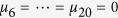
	(S16)	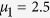 , 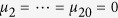

**Table 4:** Eigenvalues, cumulative contribution rates and non-centrality parameters for Scenarios (S1) to (S8).

*i*	S1–S4		S1	S2	S3	S4	S5–S8		S5	S6	S7	S8
	*λ*_*i*_	*c*_*i*_ (%)	Ω_*i*_	Ω_*i*_	Ω_*i*_	Ω_*i*_	*λ*_*i*_	*c* (%)	Ω_*i*_	Ω_*i*_	Ω_*i*_	Ω_*i*_
1	16.2	81%	4.94	0.20	0.05	0.01	4.8	24%	1.67	2.34	0.59	0.04
2	0.2	82%	0.00	4.92	1.56	0.28	0.2	28%	0.00	0.00	0.00	0.00
3	0.2	83%	0.00	0.68	0.07	0.00	0.2	32%	0.00	3.61	0.19	0.21
4	0.2	84%	0.00	1.26	2.46	0.00	0.2	36%	0.00	0.02	1.31	0.00
5	0.2	85%	0.00	1.88	3.83	0.00	0.2	40%	0.00	1.74	3.09	4.29
6	0.2	86%	0.00	0.58	2.68	17.22	0.2	44%	0.00	0.45	0.25	0.00
7	0.2	87%	0.00	0.01	0.01	0.00	0.2	48%	0.00	0.00	0.16	0.00
8	0.2	88%	0.00	0.08	0.09	0.00	0.2	52%	0.00	0.65	0.00	0.00
9	0.2	89%	0.00	0.06	0.13	0.00	0.2	56%	0.00	0.2	0.16	0.00
10	0.2	90%	0.00	0.09	0.06	0.00	0.2	60%	0.00	0.38	0.16	0.00
11	0.2	91%	0.00	0.08	0.01	0.00	0.2	64%	0.00	0.28	0.05	0.00
12	0.2	92%	0.00	0.47	0.19	0.00	0.2	68%	0.00	2.11	1.47	0.00
13	0.2	93%	0.00	0.07	0.08	0.00	0.2	72%	0.00	0.80	0.09	0.00
14	0.2	94%	0.00	0.16	0.01	0.00	0.2	76%	0.00	0.62	0.49	0.00
15	0.2	95%	0.00	0.07	0.25	0.00	0.2	80%	0.00	1.39	0.14	0.00
16	0.2	96%	0.00	2.41	0.07	0.00	0.2	84%	0.00	0.80	2.48	0.12
17	0.2	97%	0.00	0.23	0.39	0.00	0.2	88%	0.00	0.12	0.23	0.00
18	0.2	98%	0.00	0.10	0.09	0.00	0.2	92%	0.00	0.10	0.23	0.00
19	0.2	99%	0.00	0.12	0.01	0.00	0.2	96%	0.00	0.00	0.00	0.00
20	0.2	100%	0.00	2.73	0.01	1.50	0.2	100%	0.00	0.79	0.06	0.13

**Table 5:** Eigenvalues, cumulative contribution rates and non-centrality parameters for Scenarios (S9) to (S16).

*i*	S9–S12		S9	S10	S11	S12	S13–S16		S13	S14	S15	S16
	*λ*_*i*_	*c*_*i*_ (%)	Ω_*i*_	Ω_*i*_	Ω_*i*_	Ω_*i*_	*λ*_*i*_	*c* (%)	Ω_*i*_	Ω_*i*_	Ω_*i*_	Ω_*i*_
1	7.23	36.1%	5.97	1.49	0.45	0.01	1.49	7.50%	11.60	6.51	1.25	0.01
2	4.32	57.8%	0.00	2.26	1.65	0.08	1.46	14.8%	0.00	6.60	3.54	0.05
3	2.45	70.0%	0.64	0.16	2.37	0.20	1.42	21.8%	1.25	0.70	4.31	0.11
4	1.47	77.4%	0.00	0.21	1.49	0.39	1.36	28.6%	0.00	0.00	2.95	0.19
5	0.96	82.1%	0.20	0.05	0.21	0.62	1.29	35.1%	0.42	0.24	1.06	0.27
6	0.67	85.5%	0.00	0.82	0.12	0.88	1.22	14.2%	0.00	0.79	0.11	0.37
7	0.50	88.0%	0.09	0.02	0.57	1.14	1.15	47.0%	0.20	0.11	0.00	0.45
8	0.38	89.9%	0.00	0.35	0.32	1.38	1.08	52.4%	0.00	0.01	0.01	0.53
9	0.31	91.4%	0.05	0.01	0.00	1.56	1.02	57.5%	0.10	0.06	0.02	0.58
10	0.26	92.7%	0.00	0.65	0.56	1.68	0.96	62.2%	0.00	0.31	0.24	0.61
11	0.22	93.8%	0.02	0.01	1.11	1.72	0.90	66.8%	0.06	0.03	0.45	0.62
12	0.19	94.7%	0.00	0.42	0.62	1.67	0.86	71.0%	0.00	0.03	0.33	0.60
13	0.17	95.6%	0.01	0.00	0.01	1.55	0.81	75.1%	0.03	0.02	0.07	0.55
14	0.15	96.3%	0.00	0.56	0.34	1.36	0.78	79.0%	0.00	0.17	0.01	0.48
15	0.14	97.0%	0.00	0.00	0.80	1.12	0.75	82.7%	0.01	0.01	0.07	0.39
16	0.13	97.7%	0.00	0.46	0.41	0.86	0.72	86.3%	0.00	0.05	0.44	0.30
17	0.12	98.3%	0.00	0.00	0.00	0.59	0.70	89.8%	0.00	0.00	0.00	0.21
18	0.12	98.9%	0.00	0.53	0.47	0.35	0.69	93.3%	0.00	0.11	0.10	0.12
19	0.11	99.4%	0.00	0.00	0.93	0.16	0.68	96.7%	0.00	0.00	0.18	0.06
20	0.11	100%	0.00	0.49	0.46	0.04	0.67	100%	0.00	0.07	0.09	0.01
